# Comparative optimization of electric robo-taxi (eRT) and electric unmanned aerial vehicle (eUAV) systems

**DOI:** 10.1038/s41598-026-42843-y

**Published:** 2026-04-17

**Authors:** Hyeonbeen Seo, Subeen Kim, Buyong Shin, Ungki Lee

**Affiliations:** Department of Mobility Science and Engineering, Korea University, Sejong, 30019 Republic of Korea

**Keywords:** Electric robo-taxis (eRTs), Electric unmanned aerial vehicles (eUAVs), Transportation, System optimization, Energy and society, Energy science and technology, Engineering

## Abstract

This study presents a comparative optimization framework for electric robo-taxi (eRT) and electric unmanned aerial vehicle (eUAV) systems under a unified set of design and operational constraints. The objective is to evaluate the trade-off between total system cost and target travel time, providing a quantitative basis for assessing the feasibility and efficiency of both urban mobility modes. The proposed framework integrates fleet sizing, charging infrastructure design, and battery capacity optimization. The results demonstrate the differences in optimal design and cost-performance characteristics between the eRT and eUAV systems across various target travel times. The eRT system is found to be more cost-effective due to its lower infrastructure requirements, whereas the eUAV system significantly reduces travel time, offering a compelling solution for fast urban transport. The study focuses on system-level comparative analysis, excluding aspects such as real-world calibration, passenger pooling, regulatory constraints, and user behavior modeling, which are reserved for future research. This work offers insights into the design trade-offs and operational feasibility of emerging electric mobility systems under common optimization conditions.

## Introduction

Urban mobility faces increasing challenges due to traffic congestion, environmental concerns, and the need for efficient transportation solutions. The adoption of electric vehicles (EVs) mitigates dependence on fossil fuels and contributes to a reduction in greenhouse gas emissions and air pollution^1^. Electric robo-taxis (eRTs), integrating car sharing, electrification, and autonomous driving technology, are regarded as an innovative future transportation solution that will revolutionize urban transportation systems^2^. In many studies, eRTs are also referred to as shared autonomous electric vehicles (SAEVs)^3–5^. Compared to traditional taxis, eRTs can operate efficiently and safely with a smaller fleet size, significantly reducing mobility costs and wait times, while enhancing transportation accessibility and customer satisfaction^6^. Numerous recent studies have focused on eRT fleet operation and system design optimization to enhance operational efficiency and minimize system costs. Wang,^2^ proposed a multi-stage optimization strategy to optimize the location and capacity of charging infrastructures for eRT fleets, addressing issues like insufficient capacity and low utilization. Using graph theory, Monte Carlo simulation, and an improved particle swarm optimization algorithm, the strategy determines the minimum fleet size, simulates spatio-temporal charging demand, and minimizes comprehensive costs. Kim et al.,^7^ proposed a deep learning-based strategy for idle vehicle relocation, incorporating a passenger demand prediction model to improve the efficiency of fleet operations. This strategy was validated through the optimization of the eRT system design, including vehicle and charging infrastructure configurations, demonstrating substantial reductions in operational costs and customer wait times. Lee et al.,^8^ proposed a design and optimization framework for eRT system that accounts for dynamic battery degradation under varying conditions, using a semi-empirical method to predict changes based on factors like temperature, C-rate, cycle number, and depth of discharge (DOD). Results demonstrate that incorporating battery degradation into the optimization process and utilizing DOD and charging power as key design variables can significantly reduce the total system cost.

Unmanned aerial vehicles (UAVs), which are aircraft platforms that operate without the need for an onboard human pilot, can function autonomously through the integration of various sensors and microcontrollers^9^. In recent decades, UAVs have seen rapid growth in both civil and military applications^10,11^. Traditionally, most UAVs relied on internal combustion engines or hybrid propulsion systems. However, electric propulsion systems have gained significant attention due to their reduced emissions, lower noise levels, and cost-effectiveness, leading to the emergence of electric UAVs (eUAVs) powered by electric energy stored in batteries^12^. In the field of transportation, eUAVs have demonstrated significant applicability and feasibility, prompting extensive research efforts to establish eUAVs as a viable mode of transportation. Zhang et al.,^13^ explored the joint operation of coupled power and electric aviation transportation systems, focusing on dynamic en-route charging assignment for electric eUAVs to smooth power system loads while accommodating time-varying travel demands. A joint pricing scheme is proposed to minimize costs and optimize system performance, with numerical experiments demonstrating its effectiveness and benefits for integrated power and eUAV operations. Luo et al.,^14^ investigated the operating characteristics of power batteries in electric vertical take-off and landing (eVTOL) aircraft to identify factors significantly affecting battery performance and recharge mileage and derived the optimal operating conditions for eVTOL systems. Michel et al.,^15^ developed a comprehensive system-level model for eVTOL aircraft, integrating subsystem dynamics such as rotor aerodynamics, motor electro-mechanical behavior, battery dynamics, and airframe rigid body dynamics to enhance propulsion performance and optimize cruising efficiency.

However, limited research has been conducted on the optimization of eUAV systems used for passenger transportation. Furthermore, few studies have explored the comparative analysis of design optimization for eRT and eUAV systems, which are both considered promising future transportation modes, making it challenging to identify the characteristics of each mode in human transportation. This lack of comparative research hinders informed decision-making for policymakers and urban planners. Therefore, this study proposes a design framework for eRT and eUAV systems to compare the feasibility and utility of each transportation mode, providing insights into the direction of future transportation systems. In this study, the design framework for the eRT system incorporates the eRT operation model, charging station (CS) model, and eRT model, whereas the eUAV system design framework integrates the eUAV operation model, vertiport charging station (VCS) model, and eUAV model. In each system, factors affecting fleet operation—such as fleet size, charging infrastructure design, and battery capacity—serve as design variables, with passenger travel time used as a metric to assess transportation capacity. Although eRTs offer advantages such as low fleet and CS costs and an extended driving range, limitations in reducing travel time arise due to the impact of traffic and lower driving speeds on roads. In contrast, eUAVs involve higher fleet and VCS costs and a shorter flying range, but are unaffected by traffic, allowing for shorter travel time.

The remainder of this paper is constructed as follows. Section  2 presents the system design framework including testbed modeling, eRT system modeling, and eUAV system modeling. In Sect.  3, optimization formulations for eRT and eUAV systems are provided. Section  4 presents optimization results and discussion of the study. Finally, the conclusion and future directions of this study are summarized in Sect.  5.

## System design framework

In this section, system design frameworks for eRT and eUAV are presented. The eRT operation model, CS model, and eRT model constitute the eRT system, while the eUAV operation model, VCS model, and eUAV model constitute the eUAV system. However, both eRT and eUAV systems are similar in that the total system cost and passenger travel time are obtained as outputs. Before explaining the system design framework, the testbed on which the system will be applied is introduced.

### Testbed modeling

**Fig. 1:**
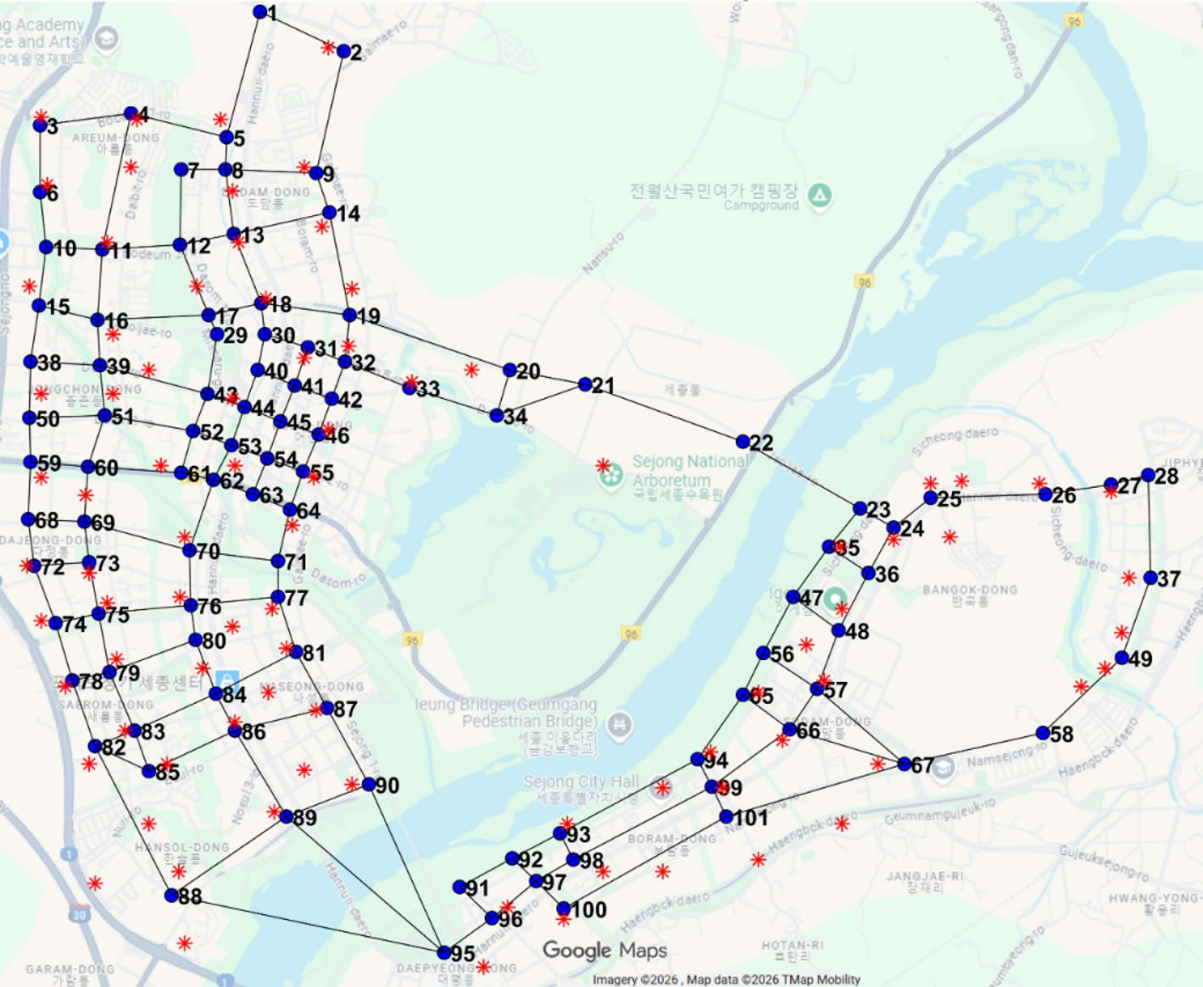
Road connections in Sejong City.

**Fig. 2:**
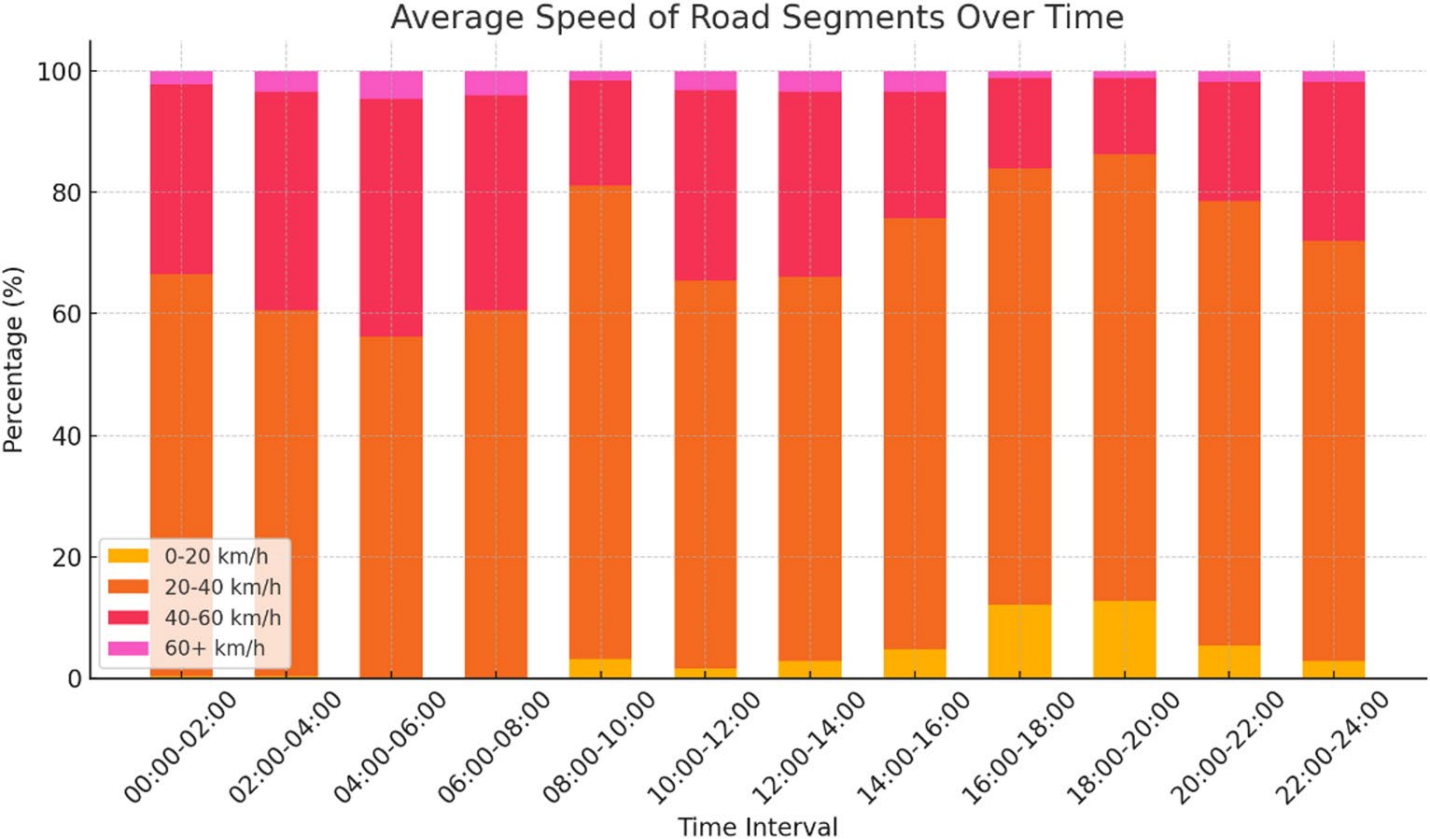
Average speed of roads over time.

In this study, Sejong City, the administrative capital of South Korea, is adopted as a testbed for the implementation of eRT and eUAV systems. Sejong City is considered a pioneering smart city leveraging advanced information and communication technology (ICT), and plans to introduce autonomous vehicles, shared mobility options, and integrated transportation systems that combine various modes of mobility^16^. Figure 1 illustrates the main road connections in Sejong City, represented by 101 nodes and 161 segments, as well as the optimal locations for CS and VCS. The real-time traffic of each road segment, collected from NAVER Map, is incorporated into the eRT simulation, and the average speed of roads over time in Sejong City is summarized in Fig. 2 (NAVER Map^17^. The average speed of roads in Sejong City is 34.8 km/h. The average speed data shows that during commuting hours, the number of road segments with traffic moving below 20 km/h increases due to higher traffic volumes. Passenger information data, including request time, origin, and destination, are stochastically generated based on urban traffic data from Sejong City (Urban Traffic Information Center^18^, which provide statistical distributions of boarding and alighting times and locations.

### Design framework for eRT system

**Fig. 3:**
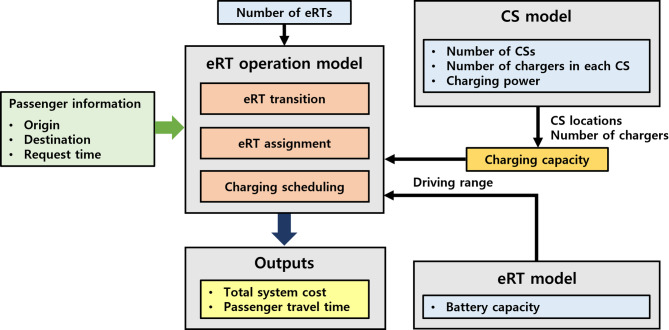
Design framework of the eRT system.

This section presents the design framework for the eRT system by introducing several models relevant to it: the eRT operation model, the CS model, and the eRT model. Figure 3 shows the design framework of the eRT system. The eRT system is comprised of three primary models: the eRT operation model, which manages eRT transition, assignment, and charging scheduling; the CS model, which determines CS locations, number of chargers, and charging power; and the eRT model, which specifies vehicle driving range. It is assumed that the eRT system operator determines the system design and that the central operating system manages the status of the eRT system and trip requests in real time. Once the system design variables are established, total system cost and passenger travel time are obtained as outputs through eRT simulation. Therefore, the key performance metrics of the system design include cost-effectiveness, which evaluates operational and infrastructure costs for economic viability, and travel time efficiency, which compares passenger transport durations in urban settings.

#### eRT operation model

**Fig. 4:**
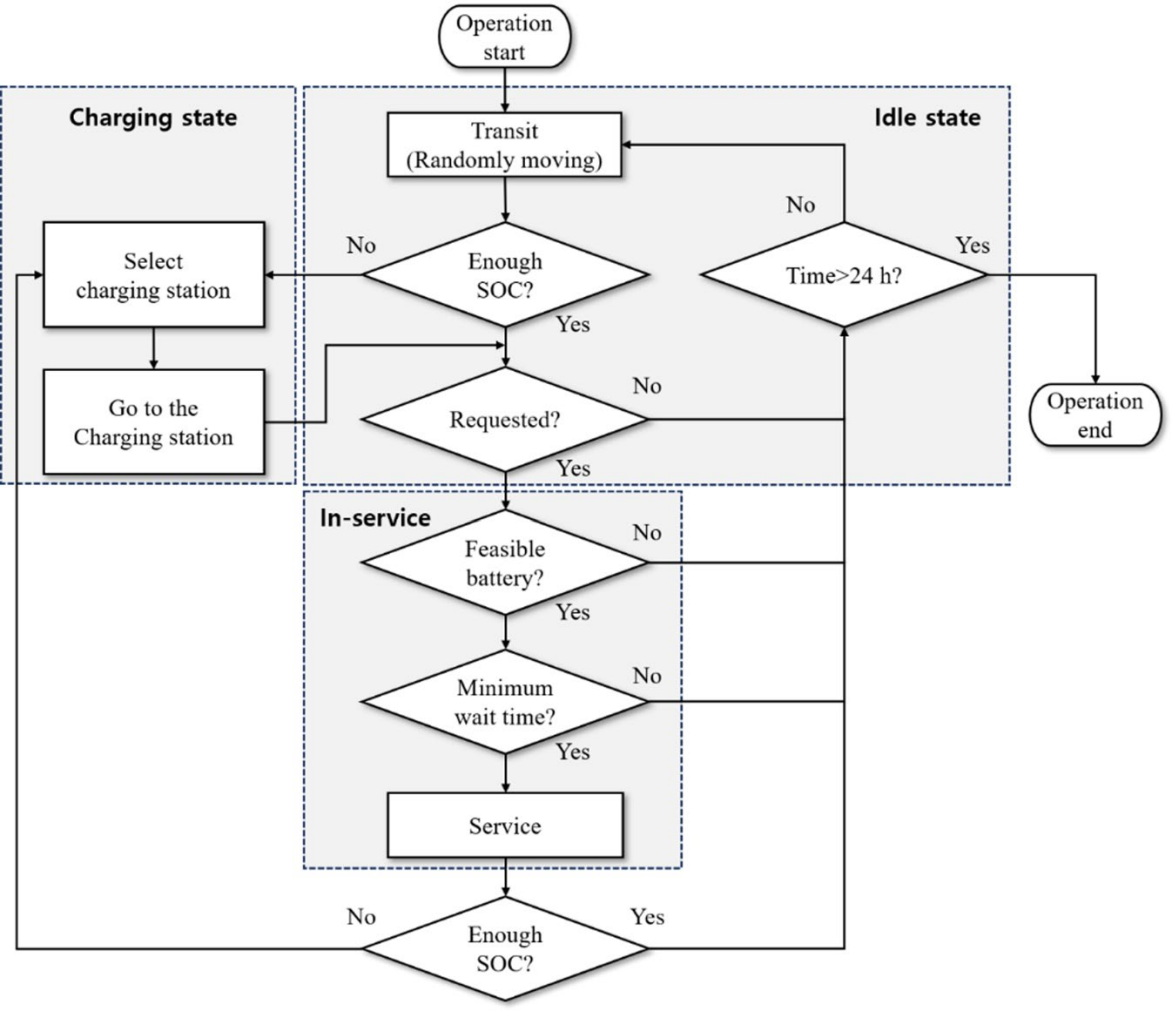
Flowchart of the eRT operation model.

The eRT operation model derives passenger travel time, which is the time taken from a passenger request to arrival at the destination, as an output through optimal eRT operation. Three states, including idle state, in-service state, and charging state, mainly constitute the state of the eRT fleet. In the idle state, the eRT is not in service and moves randomly on the road, while in the in-service state, the eRT is assigned to the passenger, indicating that the eRT is occupied. The charging state is defined as the duration encompassing both the travel time to the charging station and the actual charging process at the station. Figure 4 shows the flowchart of the eRT operation model. The initial eRT information such as initial eRT location, initial state-of-charge (SOC), and initial state are set randomly before starting the simulation. The central operating system continuously checks the battery status of each eRT and monitors the status of the CS to manage the charging schedule. If the SOC falls below a certain level, the central operating system commands the eRT to go to the nearest CS. In the event that all CSs are occupied, the CS with the shortest wait time is assigned. When a passenger request occurs, eRTs with sufficient SOC to reach the CS after service are included in the candidate vehicles. Subsequently, the eRT that minimizes passenger wait time among the candidate vehicles is selected to perform the service. The battery SOC is checked to determine whether charging is required after the service is finished, and the idle state is then started.

Several assumptions and explanations related to the eRT operation are as follows:

1. It is assumed that eRTs use fully autonomous modules.
2. Real-time traffic data in Sejong City is combined with the shortest path algorithm proposed by Dijkstra^19^ to ensure that eRT follows the shortest time path when moving to another location.
3. Passenger information is transmitted to the central operating system in real time through the passenger’s smartphone app.
4. Passenger pickup and drop-off are assumed to occur only at intersections.
5. If a passenger’s origin or destination does not exactly coincide with an intersection, the passenger is required to walk from their actual location to the nearest intersection, and the corresponding walking time is estimated assuming a walking speed of 6 km/h^20^.
6. The time required for passengers to board and disembark the eRT is assumed to be 3 min.

#### CS model

In the CS model, all nodes are treated as candidate locations for installing CSs. To identify the optimal CS configuration, a full enumeration approach is employed. For each possible number of CSs, the system optimization is performed independently to obtain the system design that minimizes the total system cost while satisfying all constraints. This procedure iteratively evaluates every feasible combination of CS counts and their corresponding candidate node locations. After examining all configurations, the CS layout that achieves the lowest total system cost is selected as the optimal solution. Since the total number of chargers is determined as the number of chargers in each CS is given, the overall charging capacity is defined by the combination of the number of CSs and chargers. This capacity directly influences the passenger travel time by limiting the number of vehicles that can be charged simultaneously. In the subsequent operation-level simulation, such capacity constraints are dynamically reflected through queuing and scheduling processes, ensuring that the impact of limited charger availability is realistically incorporated into system performance evaluation^5^^,^^21–23^. In this study, a direct-current fast charger is used and charging power is defined as a design variable. The CS model also determines CS cost based on the number of CSs and chargers: the installation cost of each charger is assumed to be $20,000^24^, with a maintenance cost of $5,500 per CS^21^, and the equipment cost of a charger according to charging power is assumed to be $520/kW^24^. The electricity cost is assumed to be 20 cents/kWh^25^.

#### eRT model

**Fig. 5:**
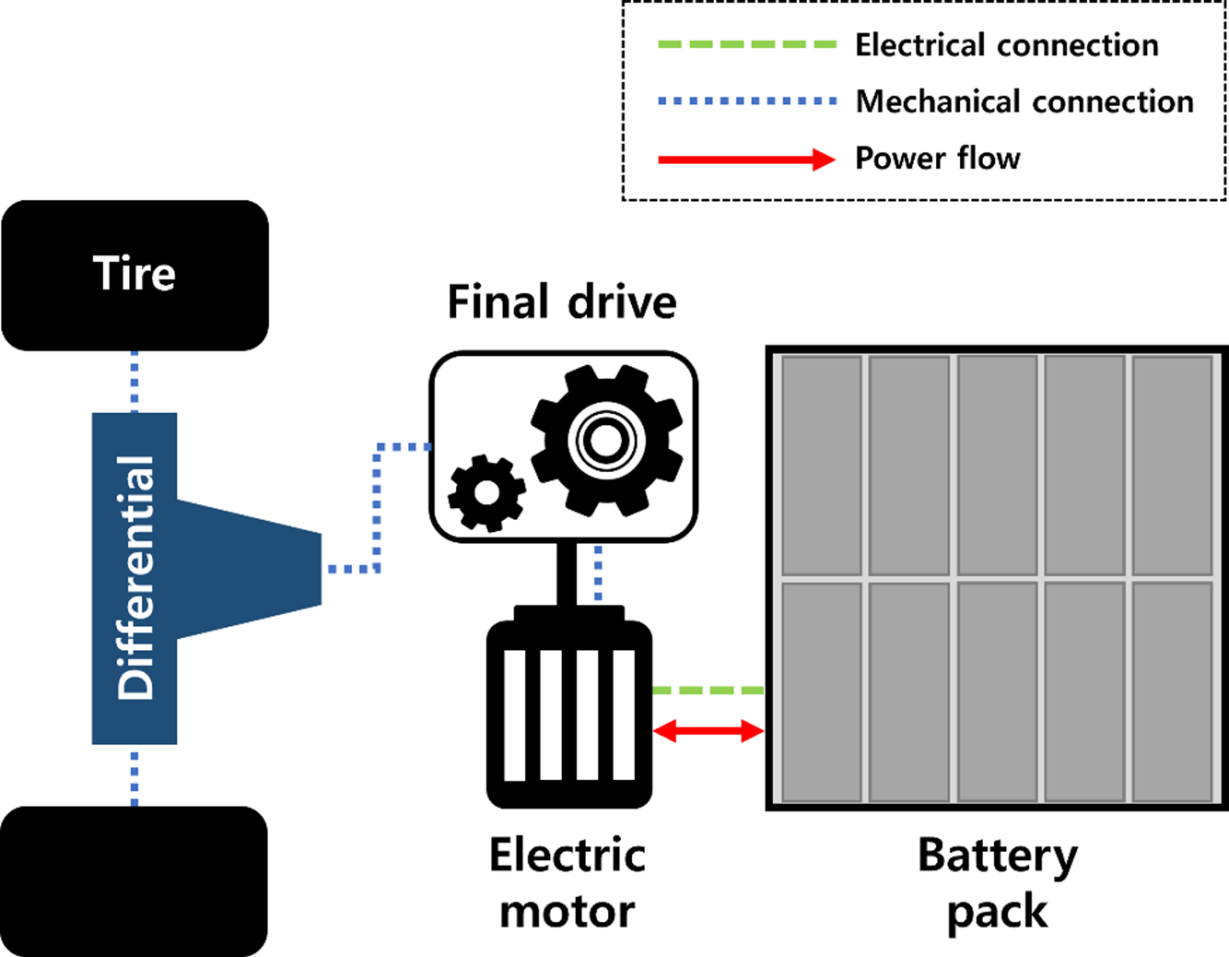
Block diagram of eRT model.

The eRT model is constructed based on the SAEV model proposed by Lee et al.,^26^. In the eRT model, the electric motor drives the wheels through the final drive and differential using electrical energy supplied by the battery pack, enabling the evaluation of vehicle performance metrics such as driving range, top speed, acceleration, and miles per gallon gasoline equivalent (MPGe). Figure 5 shows the block diagram of the eRT model, which integrates a battery pack, electric motor, motor torque control, control unit, three-phase inverter, gearbox and wheels to drive the vehicle. The capacity of the battery pack is defined as a design variable since the battery capacity directly affects the driving range of eRT. The eRT cost can be estimated as the sum of the following costs: the vehicle baseline cost is assumed to be $35,000 with reference to the average EV cost^27^, the cost of a lithium-ion battery is assumed to be $236/kWh (The Mack Institute^28^, and the cost of an autonomous module is assumed to be $10,000^29^. Further details and equations for the eRT model are available in Lee et al.,^1^ and Lee^23^.

### Design framework for eUAV system

**Fig. 6:**
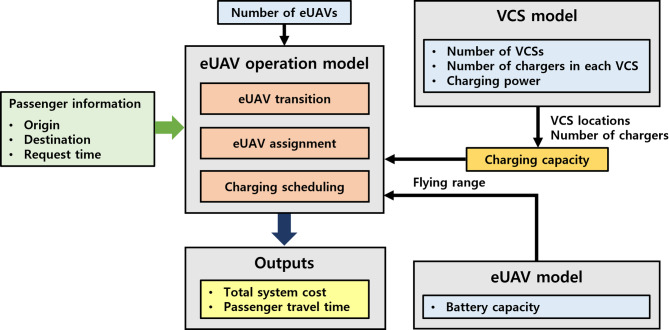
Design framework of the eUAV system.

As in Sect.  2.2, this section presents the design framework for the eUAV system along with several related models: the eUAV operation model, the vertiport model, and the eUAV model. The design framework of the eUAV system is shown in Fig. 6. The eUAV system comprises three models, similar to those used in the eRT system: the eUAV operation model, which manages eUAV transitions, assignments, and charging scheduling; the VCS model, which determines VCS locations, number of chargers, and charging power; and the eUAV model, which specifies the flying range of the eUAV. As in the eRT system, it is assumed that the eUAV operator determines the system design and that the central operating system manages the status and trip requests of the eUAV system in real time. Similarly, once the system design variables are specified, the eUAV simulation yields the total system cost and passenger travel time.

#### eUAV operation model

**Fig. 7:**
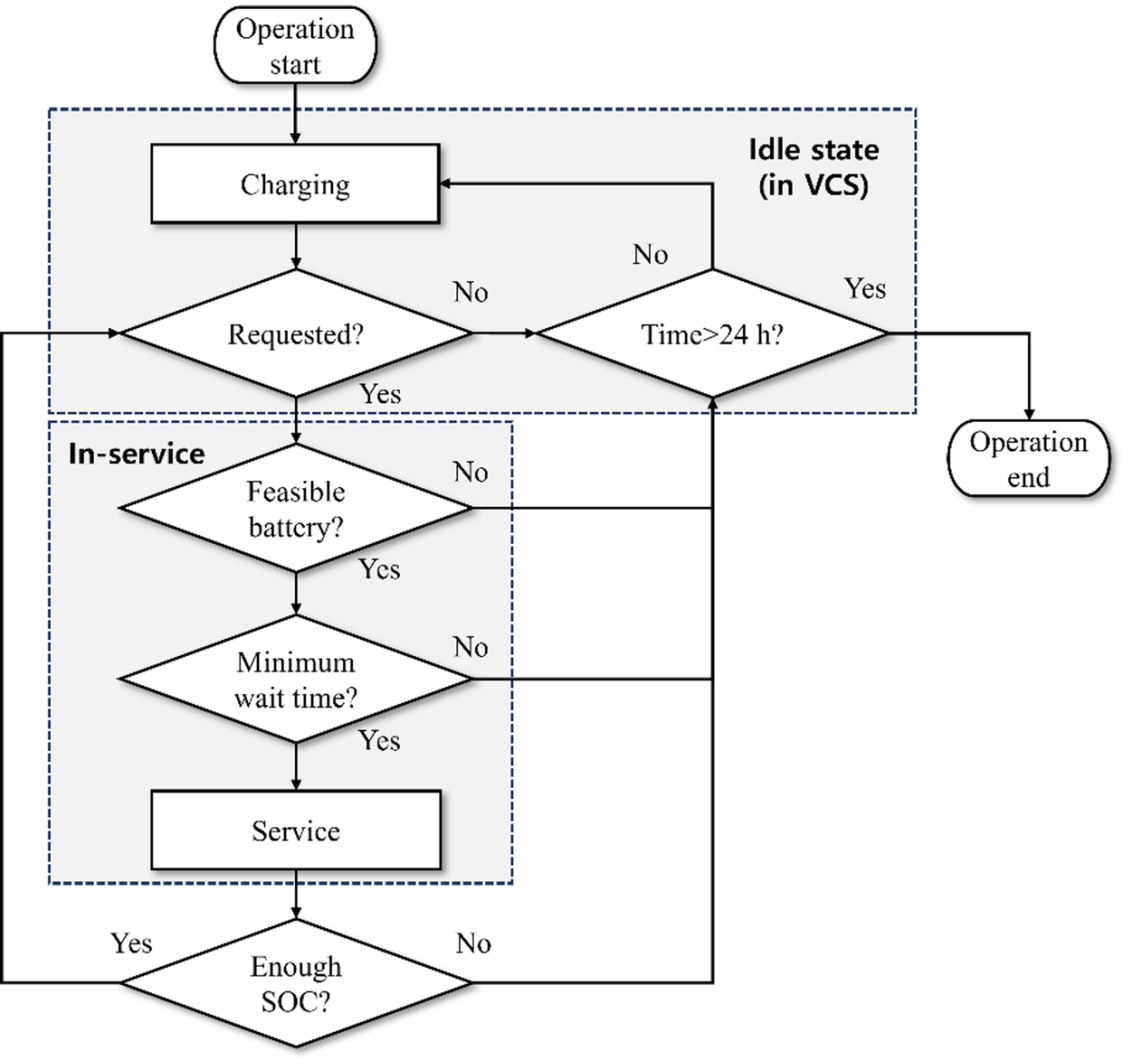
Flowchart of the eUAV operation model.

Through the eUAV operation model, eUAVs can be operated optimally, and passenger travel time can be obtained. Unlike the eRT operation model, the eUAV operation model considers only two states: idle state and in-service state. The idle state occurs at the VCS, as the eUAV remains at the vertiport when not in service. Charging occurs while the eUAV stays in the VCS. The in-service state indicates that the eUAV is assigned to a passenger. Figure 7 shows the flowchart of the eUAV operation model. The VCS location where the eUAV initially resides and the initial battery status are set randomly before starting the simulation. The central operating system continuously monitors the battery status of each eUAV and the status of the VCS to manage the charging schedule. Even after charging is complete, the eUAV remains at the VCS unless a passenger request occurs. When a passenger request occurs, the eUAV that minimizes passenger wait time, among those with sufficient battery to return to the VCS after the service, is assigned to the passenger. After completing the service, if the eUAV retains sufficient SOC and another request is generated, it is reassigned to the next service. Otherwise, it redirects to the nearest available vacant VCS to initiate the charging process in preparation for the next assignment. Passenger pickup and drop-off are assumed to occur at pre-selected high-rise buildings within Sejong City, each with an approximate height of 100 m, as shown by red asterisks in Fig. 1. The eUAV ascends to an altitude of 300 m to avoid obstacles from tall buildings and then travels directly to the building closest to the passenger’s origin or destination^30^.

Several assumptions and explanations related to the eUAV operation are as follows:

1. It is assumed that eUAVs use fully autonomous modules.
2. The passenger information used in the simulation and the process by which a passenger requests a ride are identical to those in the eRT system.
3. The passenger capacity per eUAV is limited to one person.
4. Passengers are required to walk from their actual origin or destination to the building where the eUAV takes off or lands, and the vertical travel time to reach the rooftop is estimated considering an elevator speed of 240 m/min (Hyundai Elevator^31^.
5. The climb rate of the eUAV is assumed to be 720 ft/min^32^.

#### VCS model

In the VCS model, all nodes are also considered as candidate locations for installing VCSs. Following the same procedure as in the CS model, the optimal VCS configuration is obtained by evaluating all feasible combinations of VCS counts and candidate node locations, and selecting the configuration that yields the minimum total system cost. Given the number of chargers in each VCS, the total number of chargers and the overall charging capacity are determined accordingly. Since eUAVs return to the VCS chargers after completing their service, the total number of chargers at the VCS should not be less than the total number of eUAVs. The charging space where eUAVs land and charge must be able to fully accommodate a single eUAV; therefore, one charger is installed per vertiport at each VCS. This configuration allows multiple vertiports to be installed within a single VCS to expand the overall charging capacity. The total number of chargers in each VCS thus defines the effective charging capacity, which directly affects the operational efficiency of the eUAV system by determining how many eUAVs can be simultaneously charged. In addition, unlike ground-based CSs, each VCS is subject to spatial limitations associated with landing and take-off areas, which further constrain its available charging capacity and influence system performance. As in the CS model, a direct-current fast charger is assumed to be used for charging eUAVs and charging power is defined as a design variable. The VCS cost can be calculated based on the number of VCSs and chargers. The cost of a vertiport including the installation cost is assumed to be $110,000 (Aviation Week Network^33^. The maintenance cost of a VCS and the equipment cost of a charger are assumed to be $5,500 and $520/kW, respectively, which are equivalent to those of the CS. The electricity cost is assumed to be 20 cents/kWh as in the CS model.

#### eUAV model

**Fig. 8:**
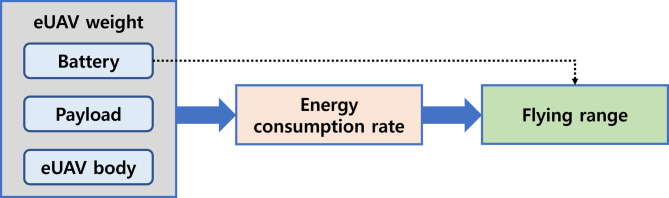
Factors affecting the flying range of an eUAV.

Electrical energy stored in the battery generates lift and thrust forces, enabling eUAVs to fly by overcoming drag forces and weight (Zhang et al., 2021). The flying range of eUAV plays an important role in eUAV simulation. Figure 8 illustrates the factors that affect the flying range of an eUAV. The flying range is determined by the energy consumption rate, which is influenced by the total takeoff weight of the eUAV, including the weight of the battery, payload, and the eUAV body. In addition, the flying range extends as the battery capacity increases; however, the energy consumption rate also rises due to the increased weight, affecting the decrease in the flying range. In this study, the energy consumption model proposed by D’Andrea^34^, which takes into account the total mass of the aircraft, flight speed, lift-to-drag (L/D) ratio, and battery power transfer efficiency, is used to calculate the flying range of the eUAV. According to the basic principles of flight, four main forces act on an aircraft: gravity acting on the total mass of the aircraft, thrust propelling the aircraft forward, lift opposing gravity, and drag opposing the forward movement. The equations and details for the eUAV model are provided in Appendix.

As the basic platform for the eUAV model, the Jetson ONE, which is a single-seat eVTOL aircraft made of aluminum-Kevlar composite structure with an empty mass of 85 kg, is referenced^35^. Powered by a motor generating a total of 102 horsepower, the Jetson ONE can reach a top speed of 101 km/h, which is assumed to be the maximum cruising speed of the eUAV employed in this study. To determine the battery mass based on the battery capacity, the specific energy density of the battery is assumed to be 250 Wh/kg^36^. Battery capacity, which can determine the flying range of an eUAV, is used as a design variable for the eUAV model. The eUAV baseline cost is assumed to be $92,000^37^. As in the eRT model, the costs of the lithium-ion battery and autonomous module are assumed to be $236/kWh and $10,000, respectively.

## Optimization framework

This section presents the optimization framework for eRT and eUAV system. Passenger transportation is carried out by integrating the models described in Sect. "System design framework", and the passenger travel time can be derived accordingly. Then, the optimization problem is formulated to minimize the total system cost and secure the constraint on passenger travel time. Under the assumption of the initial implementation of the two transportation systems, the number of passengers is set at 100 per day, and an operating period of one year is considered to calculate the operating cost. To ensure consistency in the cost evaluation, all cost-related parameters obtained from different literature sources and publication years are converted to 2025 values using the consumer price index (CPI) (Ministry of Data and Statistics^38^. The applied CPI values are 97.6 for 2017, 100 for 2020, 102.5 for 2021, 111.6 for 2023, 114.2 for 2024, and 116.5 for 2025, respectively.

### Optimization formulation for eRT system

The optimization formulation for the eRT system design can be given as^8^

1 $$\begin{gathered} {\text{ find }}{\mathbf{X}}{\mathrm{=}}\left[ {{N_{{\mathrm{eRT}}}},{N_{{\mathrm{CS}}}},{N_{{\mathrm{Charger}}}},{P_{{\mathrm{Charger}}}},B} \right] \hfill \\ {\text{ }}\mathop {{\mathrm{min}}}\limits_{{\mathbf{X}}} {\text{ Cost(}}{\mathbf{X}}{\mathrm{)}}={C_{{\mathrm{eRT}}}}+{C_{{\mathrm{CS}}}}+{C_{{\mathrm{Electricity}}}} \hfill \\ {\text{subject to }}{\mathbf{lb}} \leqslant {\mathbf{X}} \leqslant {\mathbf{ub}} \hfill \\ {\text{ }}{N_{{\mathrm{eRT}}}} \in \{ n \in {{\mathbb{Z}}^+}|5 \leqslant n \leqslant 100\} \hfill \\ {\text{ }}{N_{{\mathrm{CS}}}} \in \{ n \in {{\mathbb{Z}}^+}|1 \leqslant n \leqslant 10\} \hfill \\ {\text{ }}{N_{{\mathrm{Charger}}}} \in \{ n \in {{\mathbb{Z}}^+}|1 \leqslant n \leqslant 15\} \hfill \\ {\text{ }}{P_{{\mathrm{Charger}}}} \in \{ 2,4,...,98,100\} {\text{ (kW)}} \hfill \\ {\text{ }}B \in \{ 10,12,...,98,100\} {\text{ (kWh)}} \hfill \\ {\text{ }}\mu ({\mathbf{T}}{\mathrm{)}} \leqslant {T^{{\mathrm{Target}}}} \hfill \\ {\text{ }}{D_{{\mathrm{PU}}}}+{D_{{\mathrm{OD}}}}+{D_{{\mathrm{CS}}}} \leqslant {D_{{\mathrm{Available}}}} \hfill \\ {\text{ where }}\left[ {{\mathbf{T}},{C_{{\mathrm{eRT}}}},{C_{{\mathrm{Electricity}}}}} \right]={f_{{\mathrm{O}}{\mathrm{.eRT}}}}\left( {{N_{{\mathrm{eRT}}}},{{\mathbf{L}}_{{\mathrm{CS}}}},{N_{{\mathrm{Charger}}}},{R_{{\mathrm{Driving}}}},{T_{{\mathrm{Charging}}}}} \right) \hfill \\ {\text{ }}\left[ {{{\mathbf{L}}_{{\mathrm{CS}}}},{C_{{\mathrm{CS}}}}} \right]={f_{{\mathrm{CS}}}}\left( {{N_{{\mathrm{CS}}}},{N_{{\mathrm{Charger}}}}} \right) \hfill \\ {\text{ }}\left[ {{R_{{\mathrm{Driving}}}},{T_{{\mathrm{Charging}}}},{C_{{\mathrm{Battery}}}}} \right]={f_{{\mathrm{eRT}}}}\left( {B,{P_{{\mathrm{Charger}}}}} \right) \hfill \\ {\text{ }}{C_{{\mathrm{eRT}}}}={N_{{\mathrm{eRT}}}} \times ({C_{{\mathrm{eRT}}{\mathrm{.baseline}}}}+{C_{{\mathrm{Battery}}}}+{C_{{\mathrm{Module}}}}) \hfill \\ {\text{ }}{C_{{\mathrm{CS}}}}={N_{{\mathrm{CS}}}} \times ({N_{{\mathrm{Charger}}}} \times ({C_{{\mathrm{CS}}{\mathrm{.installation}}}}+{C_{{\mathrm{Charger}}}})+{C_{{\mathrm{CS}}{\mathrm{.maintenance}}}}) \hfill \\ {\text{ }}{T_{{\mathrm{Charging}}}}=\frac{{({S_{{\mathrm{Target}}}} - {S_{{\mathrm{Current}}}})B}}{{{P_{{\mathrm{Charger}}}}}} \hfill \\ \end{gathered}$$

where the objective is to minimize the total eRT system cost, which comprises the eRT cost, CS cost, and electricity cost; **X** is a design variable vector; ${N_{{\mathrm{eRT}}}}$, ${N_{{\mathrm{CS}}}}$, and ${N_{{\mathrm{Charger}}}}$ are the eRT fleet size, the number of CSs, and the number of chargers, respectively; ${P_{{\mathrm{Charger}}}}$ and *B* are the charger power and battery capacity, respectively; **lb** and **ub** represent the lower bound and upper bound, respectively; *µ*($\bullet$) is the mean measure; **T** denotes the travel time distribution; ${T^{{\mathrm{Target}}}}$ is the target travel time; ${D_{{\mathrm{PU}}}}$, ${D_{{\mathrm{OD}}}}$, ${D_{{\mathrm{CS}}}}$ represent the distances for pickup, origin-to-destination travel, and the nearest CS, respectively; ${D_{{\mathrm{Available}}}}$ denotes the distance that the eRT can currently travel based on its remaining battery capacity; *R*_Driving_ is the driving range; *C*_eRT_ is the eRT fleet cost, which includes the vehicle baseline cost ${C_{{\mathrm{eRT}}{\mathrm{.baseline}}}}$, battery cost ${C_{{\mathrm{Battery}}}}$, and autonomous module cost ${C_{{\mathrm{Module}}}}$; ${C_{{\mathrm{Electricity}}}}$ is the electricity cost; ${{\mathbf{L}}_{{\mathrm{CS}}}}$ and ${T_{{\mathrm{Charging}}}}$ denote the CS location vector and the time taken to charging, respectively; *C*_CS_ represents the CS cost, which includes the installation cost ${C_{{\mathrm{CS}}{\mathrm{.installation}}}}$, maintenance cost ${C_{{\mathrm{CS}}{\mathrm{.maintenance}}}}$, and charger equipment cost ${C_{{\mathrm{Charger}}}}$; ${f_{{\mathrm{O}}{\mathrm{.eRT}}}}$, ${f_{{\mathrm{CS}}}}$, and ${f_{{\mathrm{eRT}}}}$ are the eRT operation, CS, and eRT models, respectively; and ${S_{{\mathrm{Target}}}}$ and ${S_{{\mathrm{Current}}}}$ are the target and current SOC, respectively. Table 1 shows the design variables and their bounds for eRT system design. In this study, both the charging power and battery capacity are defined as discrete design variables to reflect practical implementation constraints. The charging power levels are selected to reflect public charging conditions, specifically Levels II and III, representing standard semi-fast and fast charging rates used in commercial charging infrastructure^39^. Similarly, the battery capacity values are discretized by referencing commercially electric vehicle specifications, ensuring that the optimization results remain realistic and practically feasible within the range of existing technologies^40^. In the optimization process, the charging power is varied from 2 kW to 100 kW, and the battery capacity is varied from 10 kWh to 100 kWh, both in increments of 2, to represent discrete and practically implementable design levels.

**Table 1:** Design variables and their bounds for eRT system design.

Design variable	Lower bound	Upper bound
eRT fleet size	5	100
Number of CSs	1	10
Number of chargers in each CS	1	15
Charging power	2 kW	100 kW
Battery capacity	10 kWh	100 kWh

### Optimization formulation for eUAV system

The optimization formulation for the eUAV system design can be given as

2 $$\begin{gathered} {\text{ find }}{\mathbf{X}}{\mathrm{=}}\left[ {{N_{{\mathrm{eUAV}}}},{N_{{\mathrm{VCS}}}},{N_{{\mathrm{Charger}}}},{P_{{\mathrm{Charger}}}},B} \right] \hfill \\ {\text{ }}\mathop {{\mathrm{min}}}\limits_{{\mathbf{X}}} {\text{ Cost(}}{\mathbf{X}}{\mathrm{)}}={C_{{\mathrm{eUAV}}}}+{C_{{\mathrm{VCS}}}}+{C_{{\mathrm{Electricity}}}} \hfill \\ {\text{subject to }}{\mathbf{lb}} \leqslant {\mathbf{X}} \leqslant {\mathbf{ub}} \hfill \\ {\text{ }}{N_{{\mathrm{eUAV}}}} \in \{ n \in {{\mathbb{Z}}^+}|1 \leqslant n \leqslant 50\} \hfill \\ {\text{ }}{N_{{\mathrm{VCS}}}} \in \{ n \in {{\mathbb{Z}}^+}|1 \leqslant n \leqslant 10\} \hfill \\ {\text{ }}{N_{{\mathrm{Charger}}}} \in \{ n \in {{\mathbb{Z}}^+}|1 \leqslant n \leqslant 15\} \hfill \\ {\text{ }}{P_{{\mathrm{Charger}}}} \in \{ 2,4,...,98,100\} {\text{ (kW)}} \hfill \\ {\text{ }}B \in \{ 10,12,...,98,100\} {\text{ (kWh)}} \hfill \\ {\text{ }}\mu ({\mathbf{T}}{\mathrm{)}} \leqslant {T^{{\mathrm{Target}}}} \hfill \\ {\text{ }}{D_{{\mathrm{PU}}}}+{D_{{\mathrm{OD}}}}+{D_{{\mathrm{VCS}}}} \leqslant {D_{{\mathrm{Available}}}} \hfill \\ {\text{ }}{N_{{\mathrm{VCS}}}} \times {N_{{\mathrm{Charger}}}} \geqslant {N_{{\mathrm{eUAV}}}} \hfill \\ {\text{ where }}\left[ {{\mathbf{T}},{C_{{\mathrm{eUAV}}}},{C_{{\mathrm{Electricity}}}}} \right]={f_{{\mathrm{O}}{\mathrm{.eUAV}}}}\left( {{N_{{\mathrm{eUAV}}}},{{\mathbf{L}}_{{\mathrm{VCS}}}},{N_{{\mathrm{Charger}}}},{R_{{\mathrm{Flying}}}},{T_{{\mathrm{Charging}}}}} \right) \hfill \\ {\text{ }}\left[ {{{\mathbf{L}}_{{\mathrm{VCS}}}},{C_{{\mathrm{VCS}}}}} \right]={f_{{\mathrm{VCS}}}}\left( {{N_{{\mathrm{VCS}}}},{N_{{\mathrm{Charger}}}}} \right) \hfill \\ {\text{ }}\left[ {{R_{{\mathrm{Flying}}}},{T_{{\mathrm{Charging}}}},{C_{{\mathrm{Battery}}}}} \right]={f_{{\mathrm{eUAV}}}}\left( {B,{P_{{\mathrm{Charger}}}}} \right) \hfill \\ {\text{ }}{C_{{\mathrm{eUAV}}}}={N_{{\mathrm{eUAV}}}} \times ({C_{{\mathrm{eUAV}}{\mathrm{.baseline}}}}+{C_{{\mathrm{Battery}}}}+{C_{{\mathrm{Module}}}}) \hfill \\ {\text{ }}{C_{{\mathrm{VCS}}}}={N_{{\mathrm{VCS}}}} \times ({N_{{\mathrm{Charger}}}} \times ({C_{{\mathrm{VCS}}{\mathrm{.installation}}}}+{C_{{\mathrm{Charger}}}})+{C_{{\mathrm{VCS}}{\mathrm{.maintenance}}}}) \hfill \\ {\text{ }}{T_{{\mathrm{Charging}}}}=\frac{{({S_{{\mathrm{Target}}}} - {S_{{\mathrm{Current}}}})B}}{{{P_{{\mathrm{Charger}}}}}} \hfill \\ \end{gathered}$$

where the objective is to minimize the total eUAV system cost, consisting of the eUAV cost, VCS cost, and electricity cost; ${N_{{\mathrm{eUAV}}}}$, and ${N_{{\mathrm{VCS}}}}$ are the eUAV fleet size and the number of VCSs, respectively; ${D_{{\mathrm{VCS}}}}$ is the distance to the nearest VCS; ${R_{{\mathrm{Flying}}}}$ is the flying range; ${C_{{\mathrm{eUAV}}}}$ and ${C_{{\mathrm{eUAV}}{\mathrm{.baseline}}}}$ are the eUAV fleet cost and eUAV baseline cost, respectively; ${{\mathbf{L}}_{{\mathrm{VCS}}}}$ is the VCS location vector; ${C_{{\mathrm{VCS}}}}$, ${C_{{\mathrm{VCS}}{\mathrm{.installation}}}}$, and ${C_{{\mathrm{VCS}}{\mathrm{.maintenance}}}}$ are the VCS cost, VCS installation cost, and VCS maintenance cost, respectively; and ${f_{{\mathrm{O}}{\mathrm{.eUAV}}}}$, ${f_{{\mathrm{VCS}}}}$, and ${f_{{\mathrm{eUAV}}}}$ are the eUAV operation, VCS, and eUAV models, respectively. Table 2 shows the design variables and their bounds for eUAV system design. Similar to the eRT system, both the charging power and battery capacity are defined as discrete design variables, with charging power varied from 2 kW to 100 kW and battery capacity varied from 10 kWh to 100 kWh, both in increments of 2.

**Table 2:** Design variables and their bounds for eUAV system design.

Design variable	Lower bound	Upper bound
eUAV fleet size	1	50
Number of VCSs	1	10
Number of chargers in each VCS	1	15
Charging power	2 kW	100 kW
Battery capacity	10 kWh	100 kWh

## Results and discussion

**Fig. 9:**
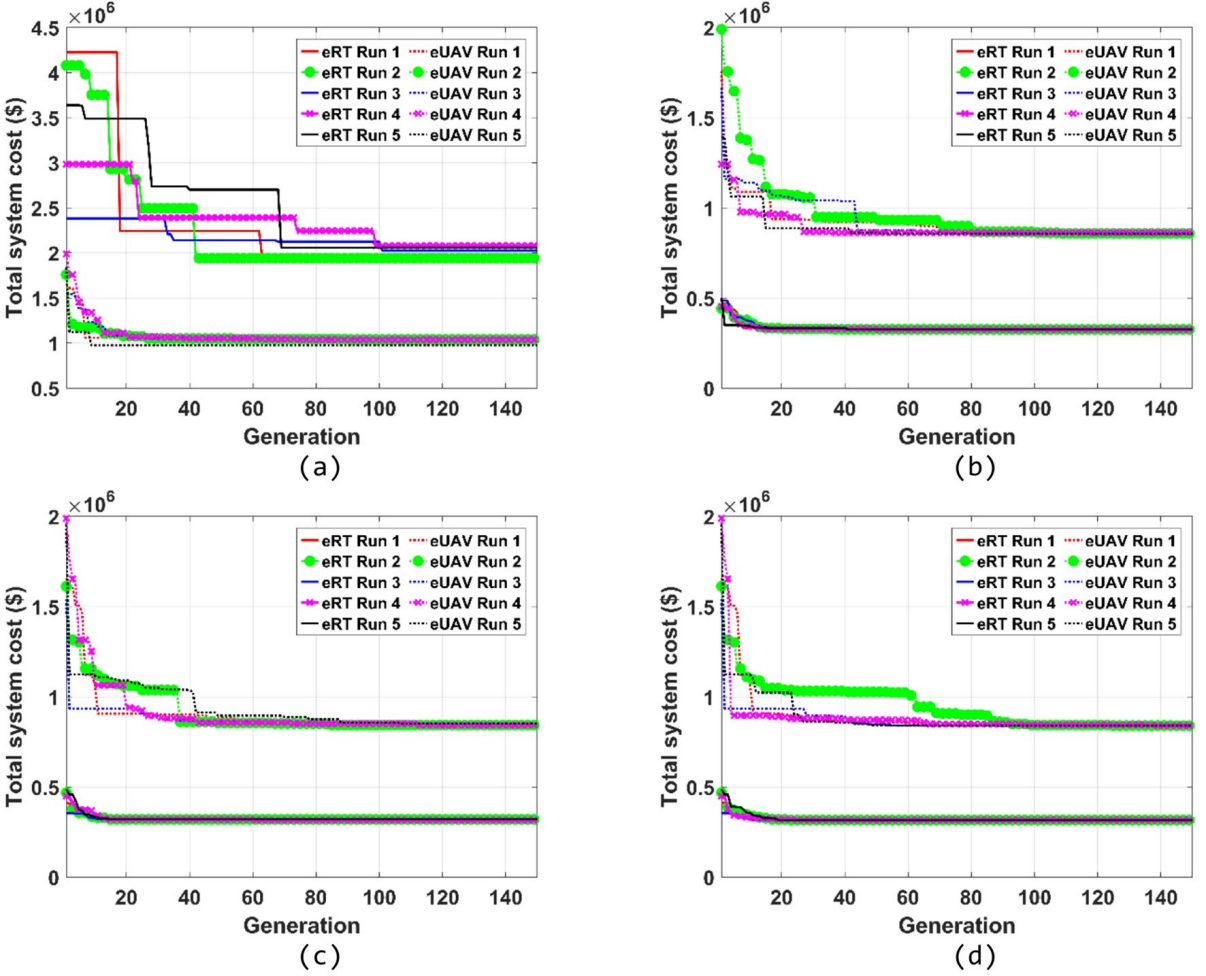
Convergence curves of the optimization results for eRT and eUAV systems. (**a**) Target travel time of 21 min, (**b**) Target travel time of 24 min, (**c**) Target travel time of 27 min, (**d**) Target travel time of 30 min.

This section presents optimization results of eRT and eUAV system designs obtained according to various target travel times. Genetic algorithm (GA) is employed to solve the optimization problems presented in Eqs. (1) and (2). The parameters used in the GA are as follows: elitism is applied such that 5% of the population is retained as elite individuals and passed to the next generation; the mutation and crossover rates are set to 1% and 80%, respectively; the population size and the maximum number of generations are set to 300 and 150, respectively; the algorithm terminates when the average relative change in the best fitness value over 50 consecutive generations falls below the predefined threshold of 1 × 10⁻⁶; and a different random seed is used for each GA trial to ensure reproducibility while maintaining stochastic diversity across runs. The average computation time required for one round of optimization is 8 h using a standard workstation (AMD Ryzen Threadripper 7980X CPU @ 128.0 GB RAM and 3.20 GHz). The optimal system designs and outcomes obtained by applying target travel times of 21, 24, 27, and 30 min are summarized in Tables 3, 4 and 5, and 6. All system designs employing eRTs and eUAVs satisfy the travel time constraints, indicating that optimization has been performed appropriately. To ensure the global optimum, the optimization is performed five times, and the resulting convergence curves are presented in Fig. 9. Except for the case with a target travel time of 21 min, the convergence trends under all other conditions remain highly consistent, confirming the stability and reliability of the GA-based optimization framework.

**Table 3:** Optimal system designs and outcomes (target travel time of 21 min).

		eRT	eUAV
Decision variables	Fleet size	26	2
	Number of CSs (VCSs)	3	1
	Node ID of CSs (VCSs)	48, 76, 81	64
	Number of chargers in each CS (VCS)	1	3
	Charging power	88 kW	98 kW
	Battery capacity	32 kWh	42 kWh
Specification	Driving (flying) range	183.6 km	27.1 km
Cost	Total system cost	$1,940,817	$904,954
	Fleet cost	$1,465,603	$256,095
	CS (VCS) cost	$249,526	$529,002
	Electricity cost per day	$627	$333
Fleet operation	Full charging time	21.8 min	25.7 min
	Mean fleet service distance per day	677.9 km	526.9 km
Service	Mean travel time	21.0 min	20.4 min

**Table 4:** Optimal system designs and outcomes (target travel time of 24 min).

		eRT	eUAV
Decision variables	Fleet size	5	2
	Number of CSs (VCSs)	1	1
	Node ID of CSs (VCSs)	43	64
	Number of chargers in each CS (VCS)	1	3
	Charging power	20 kW	92 kW
	Battery capacity	12 kWh	22 kWh
Specification	Driving (flying) range	71.7 km	18.7 km
Cost	Total system cost	$325,576	$853,884
	Fleet cost	$253,677	$244,827
	CS (VCS) cost	$41,981	$518,098
	Electricity cost per day	$83	$253
Fleet operation	Full charging time	36.0 min	14.3 min
	Mean fleet service distance per day	486.5 km	526.9 km
Service	Mean travel time	23.9 min	24.0 min

**Table 5:** Optimal system designs and outcomes (target travel time of 27 min).

		eRT	eUAV
Decision variables	Fleet size	5	2
	Number of CSs (VCSs)	1	1
	Node ID of CSs (VCSs)	56	64
	Number of chargers in each CS (VCS)	1	3
	Charging power	14 kW	86 kW
	Battery capacity	12 kWh	22 kWh
Specification	Driving (flying) range	71.7 km	18.7 km
Cost	Total system cost	$316,742	$842,980
	Fleet cost	$253,677	$244,827
	CS (VCS) cost	$38,346	$507,193
	Electricity cost per day	$69	$253
Fleet operation	Full charging time	51.4 min	15.3 min
	Mean fleet service distance per day	401.9 km	526.9 km
Service	Mean travel time	27.0 min	26.8 min

**Table 6:** Optimal system designs and outcomes (target travel time of 30 min).

		eRT	eUAV
Decision variables	Fleet size	5	2
	Number of CSs (VCSs)	1	1
	Node ID of CSs (VCSs)	18	64
	Number of chargers in each CS (VCS)	1	3
	Charging power	14 kW	84 kW
	Battery capacity	12 kWh	22 kWh
Specification	Driving (flying) range	71.7 km	18.7 km
Cost	Total system cost	$315,913	$839,345
	Fleet cost	$253,677	$244,827
	CS (VCS) cost	$38,346	$503,558
	Electricity cost per day	$66	$253
Fleet operation	Full charging time	51.4 min	15.7 min
	Mean fleet service distance per day	388.4 km	526.9 km
Service	Mean travel time	29.2 min	28.1 min

**Fig. 10:**
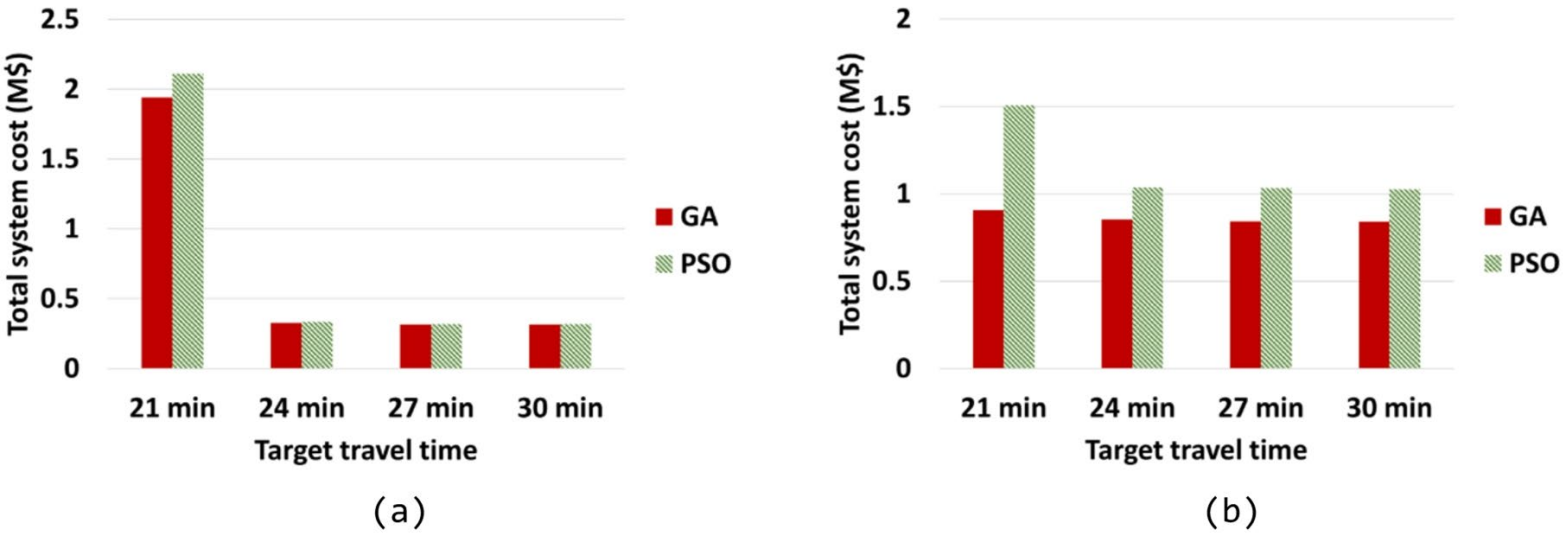
Comparison of total system cost between GA and PSO. (**a**) eRT system, (**b**) eUAV system.

To verify the performance of the baseline optimization method, GA, the particle swarm optimization (PSO) is employed for comparison under identical computational conditions. The runtime of the GA is used as the time limit for a fair comparison, and the minimum total system cost obtained from the five PSO runs is compared with that achieved by the GA. As shown in the comparison results in Fig. 10, the GA consistently outperforms the PSO, achieving a lower total system cost within the same computational time. These findings confirm that the GA serves as a robust and reliable baseline optimization method for solving the computationally complex multi-variable design problem considered in this study. When the target travel time falls below 21 min, no feasible solution exists for the eRT system. Therefore, only the results and optimal designs of the eUAV system, obtained by applying target travel times of 15 and 18 min, are presented in Table 7. All optimized eRT and eUAV system designs satisfy the travel time constraints, confirming the validity of the optimization results.

**Table 7:** Optimal designs and outcomes of eUAV system (target travel time of 15 and 18 min).

		Target travel time	
		15 min	18 min
Decision variables	Fleet size	3	3
	Number of CSs (VCSs)	2	2
	Node ID of CSs (VCSs)	53, 57	53, 57
	Number of chargers in each CS (VCS)	2	2
	Charging power	64 kW	56 kW
	Battery capacity	34 kWh	24 kWh
Specification	Driving (flying) range	24.3 km	19.8 km
Cost	Total system cost	$1,105,322	$1,065,266
	Fleet cost	$377,381	$368,930
	CS (VCS) cost	$627,219	$607,833
	Electricity cost per day	$280	$246
Fleet operation	Full charging time	31.9 min	25.7 min
	Mean fleet service distance per day	326.7 km	331.3 km
Service	Mean travel time	14.9 min	17.9 min

Optimization results can be analyzed in terms of (a) total system cost, (b) travel time, (c) CS/VCS designs, (d) eRT/eUAV designs, and (e) fleet operation as follows.

**Fig. 11:**
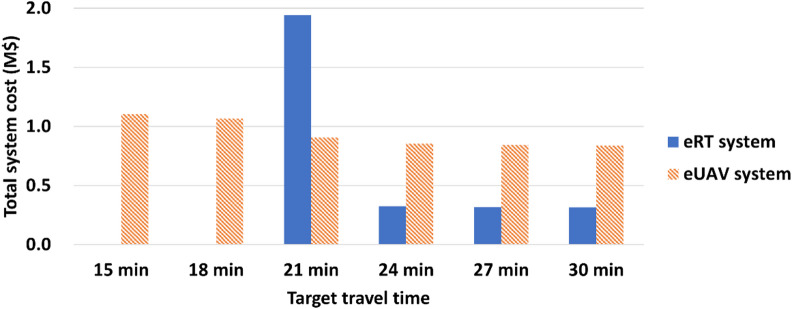
Comparison of the total system cost according to target travel time.

**Fig. 12:**
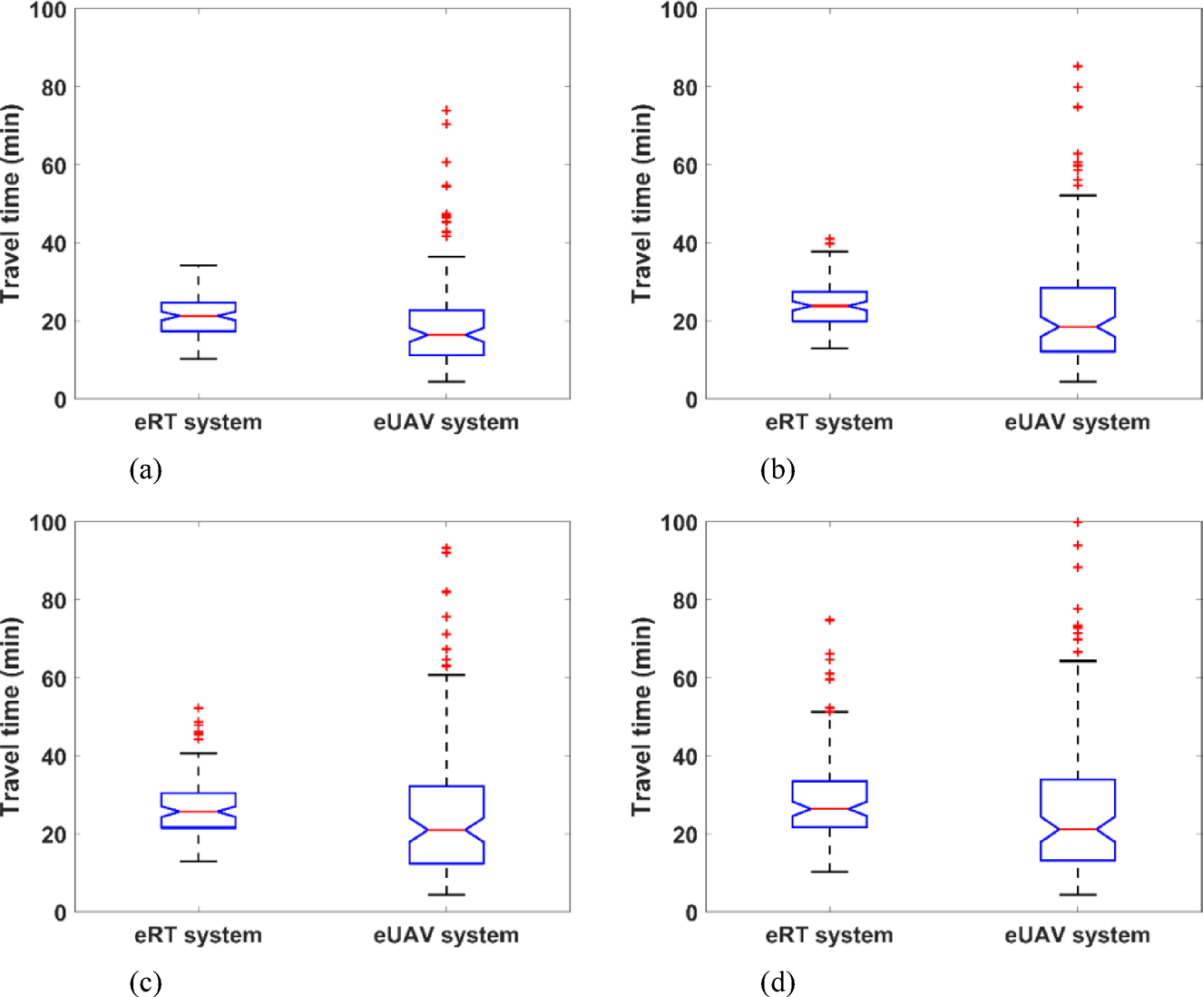
Boxplots comparing the travel time distributions of eRT and eUAV systems. (**a**) Target travel time of 21 min (**b**) Target travel time of 24 min, (**c**) Target travel time of 27 min, (**d**) Target travel time of 30 min.

- When the target travel time is high, the total system cost of the eRT system is significantly lower than that of the eUAV system. The comparison of the total system cost according to target travel time is shown in Fig. 11. However, the total system cost of the eRT system increases rapidly as the target travel time decreases, and the total system cost of the eRT system and the eUAV system becomes reversed as the target travel time decreases to 21 min. In the eUAV system, in contrast, the total system cost increases relatively modestly as the target travel time decreases. When the target travel time decreases from 30 min to 21 min, the increase rate of the total system cost of the eRT system (166.4%) is much greater than that of the eUAV system (2.6%). In addition, it is unable to derive eRT system designs that achieve travel times less than 21 min. In the eUAV system, the total system cost remains relatively stable even when the target travel time falls below 21 min, and feasible designs that satisfy such low target travel times can still be derived. In conclusion, the eRT system can be cost-effective in the existing transportation system where slow travel is allowed (high target travel time), while the eUAV system has clear potential in the future transportation system where fast travel is desired (low target travel time).

(b) While the eUAV system demonstrates a greater incidence of early arrivals than the eRT system, it is also more prone to delayed travel times. Figure 12 presents boxplots comparing the travel time distributions of eRT and eUAV systems for target travel times of 21, 24, 27, and 30 min, while summary statistics of these distributions are provided in Table 8. In each boxplot, the whiskers extend to the smallest and largest values that lie within 1.5 times the interquartile range below the first quartile and above the third quartile, respectively. Observations beyond these limits are treated as outliers and are indicated by discrete markers. The lower percentiles of the eUAV travel time distribution (i.e., shorter travel times) are more concentrated than those of the eRT system, indicating a higher frequency of early arrivals. On average, the median travel time of the eUAV system is 20.9% shorter than that of the eRT system. However, the variability in travel time is significantly greater in the eUAV system, as evidenced by the wider interquartile ranges and the presence of numerous outliers in the upper tail of the distribution. On average, the 90th percentile travel time of the eUAV system is 54.1% higher than that of the eRT system. This pronounced long-tail behavior indicates a potential risk of extreme delays in certain eUAV operations. In contrast, the eRT system demonstrates more consistent performance, with tighter distributions and fewer extreme cases. On average, the standard deviation of travel time in the eUAV system is 142.0% higher than that of the eRT system. These results imply a trade-off between shorter average travel times and greater variability in the eUAV system, highlighting the need for reliability management in aerial mobility services.

**Table 8:** Summary statistics of travel time distributions (min)

	Target travel time	Median	Standard deviation	First quartile Q1	Third quartile Q3	Interquartile range
eRT system	21	21.3	4.9	17.4	24.7	7.3
	24	23.9	6.1	19.9	27.4	7.5
	27	25.7	8	21.6	30.5	8.9
	30	26.4	12.1	21.7	33.5	11.8
eUAV system	21	16.4	14	11.2	22.7	11.5
	24	18.5	16.8	12.1	28.5	16.4
	27	21	19.3	12.4	32.2	19.8
	30	21.2	20.6	13.2	33.9	20.7

**Fig. 13:**
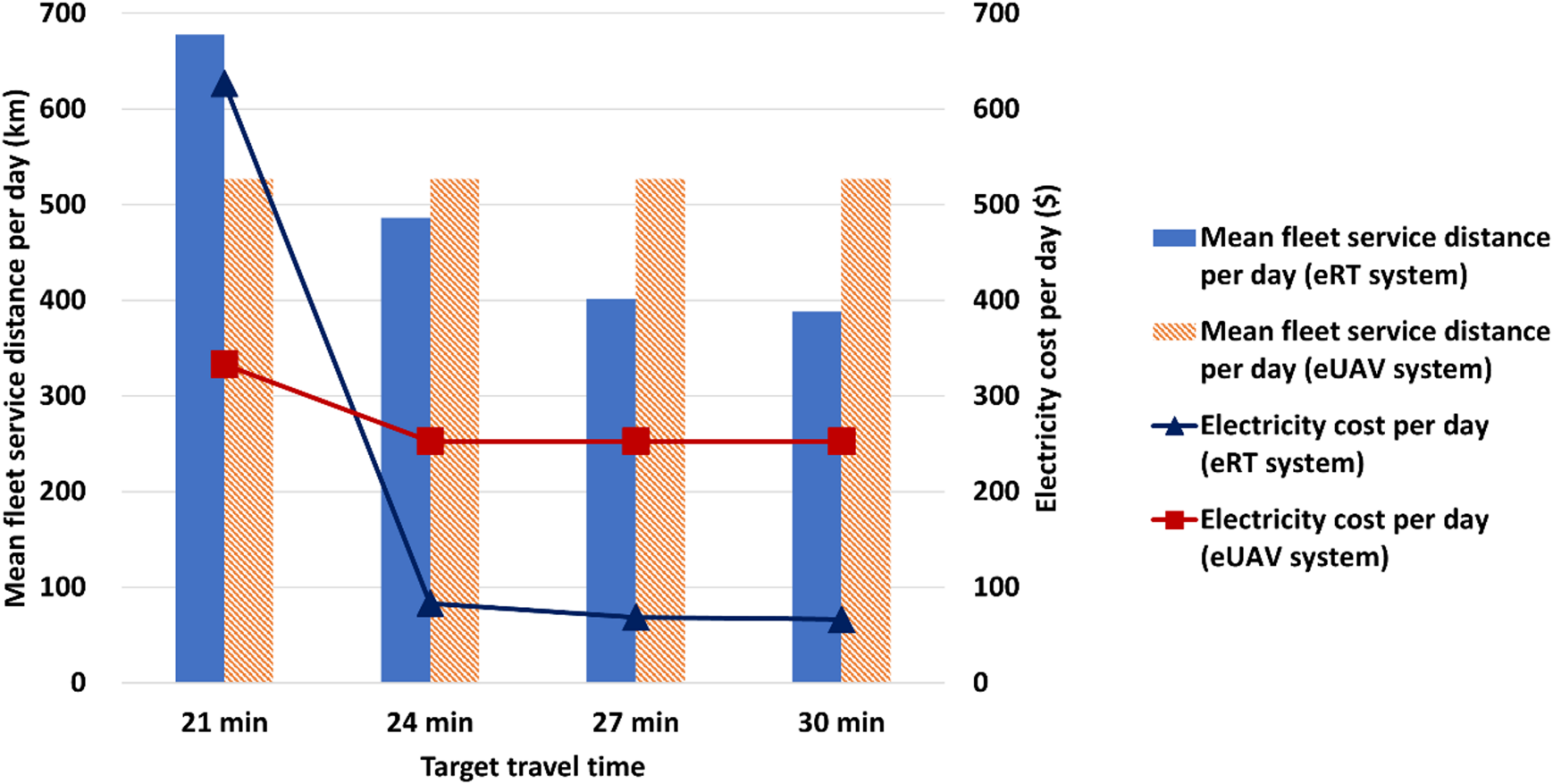
Comparison of mean fleet service distance per day and daily electricity cost between eRT and eUAV systems.

(c) In the eUAV system, a dedicated space for the eUAV to stay regardless of charging should be secured, requiring a large number of VCSs and chargers despite the high cost. On the other hand, in the eRT system, fewer CSs and chargers are required compared to the eUAV system, as eRTs only need to visit the CS for charging. This can lead to significant differences in CS and VCS costs. For target travel times of 21, 24, 27, and 30 min, the average CS and VCS costs are $92,050 and $514,463, respectively. Additionally, the variation in the total number of chargers according to the target travel time differs between the two systems. In the eRT system, the charger count shows little change down to 24 min but increases sharply at 21 min as additional charging capacity becomes necessary to maintain the reduced travel time. In contrast, in the eUAV system, the number of chargers remains constant from 30 to 21 min but increases under lower target travel times to accommodate higher service demand. In the eRT system, a CS design in which CSs are spread across several locations is derived to evenly accommodate the widely distributed eRTs, whereas a centralized VCS design that can reduce maintenance cost is preferred in eUAV system because eUAVs have little restrictions on movement, allowing easy access to VCS regardless of location. The CS locations vary, whereas the VCS locations remain unchanged from 30 to 21 min. This is because eUAVs are not affected by road layout or traffic conditions, while eRTs are influenced by both; as charging power and battery capacity change, the optimal CS locations can shift accordingly. From the perspective of charging power, the eUAV system generally exhibits higher charging power than the eRT system, mainly because of the high cost of VCSs, which makes it more economical to increase charger power rather than the number of VCSs. As the target travel time decreases, both eRT and eUAV systems show an overall increase in charging power to shorten the charging duration, in particular, the eRT system experiences a sharp rise at 21 min as additional charging capacity becomes essential to maintain the reduced travel time.
(d) The number of eUAV fleets is much smaller than the number of eRT fleets, indicating that eUAVs have higher transport capabilities compared to eRTs. The battery capacity of eUAV tends to be larger than that of eRT. This can be explained by the fact that the flying range of eUAVs is shorter than the driving range of eRTs for the same battery capacity. This is also intended to ensure that eUAV systems with smaller fleet sizes have sufficient standby fleets with adequate battery capacity to immediately pick up passengers. The battery capacity increases in both eRT and eUAV systems as the target travel time decreases to 21 min. In the eUAV system, however, when the target travel time further decreases to 18 min, the fleet size and the number of chargers increase, resulting in a reduction in both battery capacity and charging power. This adjustment reflects the strategy to balance charging infrastructure and onboard energy storage for shorter travel time conditions.
(e) The service distance of eRT tends to increase as the target travel time decreases. This is because the time eRTs spend traveling on the road increases due to the increase in charging power. Figure 13 presents the comparison between eRT and eUAV systems in terms of mean fleet service distance per day and daily electricity cost. In the case of eUAV, as there is no random movement as in eRT, the same service distance of 526.9 km is always achieved regardless of system design. For target travel times of 21, 24, 27, and 30 min, the average service distance of eRT per day is 488.7 km, which is 7.3% (38.2 km) lower than that of eUAV. Because the service distance of eUAV includes take-off and landing segments, eUAV system exhibits a slightly higher average service distance compared to eRT system, even though the travel paths of eUAVs are more direct. However, when the target travel time decreases to 21 min, the service distance of the eRT system increases sharply and surpasses that of the eUAV system due to the significantly increased fleet size. In terms of electricity cost, eRT is more efficient than eUAV. Even though the service distance of eUAV is always constant, the electricity cost per day can be varied because the flying range per kWh depends on the battery capacity. For target travel times of 21, 24, 27, and 30 min, the average electricity cost per day of eRT is $211, which is 29.1% ($61) lower than that of eUAV. However, when the target travel time decreases to 21 min, the electricity cost per day of the eRT system increases sharply and surpasses that of the eUAV system due to the significantly increased total service distance and reduced range per kWh.

Several parametric studies have been conducted to obtain insightful observations. Since limited research has been focused on eUAV system design, parametric studies varying parameters related to the eUAV system are mainly investigated. The results of the parametric studies are compared with the optimization results obtained for the target travel time of 21 min.

1. *Changes in eUAV weight*. Since the weight of the eUAV to be used in the future transportation system cannot be accurately specified, it is necessary to examine how the system design changes when the weight of the eUAV changes. To this end, optimization is carried out for the case when the body weight of the currently given eUAV is doubled. The optimization results indicate that the eUAV fleet size and VCS design remain unchanged, while the battery capacity increases by 19.0% (8 kWh), resulting in a 5.6% ($50,405) increase in the total system cost. Despite the larger battery, the flying range decreases from 27.1 km to 23.9 km. To compensate for this reduction and maintain service feasibility, the charging power increases by 2.0% (2 kW). These results show that increases in eUAV weight adversely affect flight performance, underscoring the importance of weight reduction for enhancing operational efficiency and overall system profitability.
2. *Changes in L/D ratio*. The L/D ratio defined as the ratio of lift to drag is affected by the body shape of the eUAV as well as the propeller shape^41^. To assess the impact of the L/D ratio, optimization is performed for a scenario where the L/D ratio is halved. This has an equivalent effect to reducing the battery power transfer efficiency by 50%. The optimization results show that the battery capacity increases by 85.7% (36 kWh), while the flying range increases by only 29.5% (8.0 km), indicating that reducing the L/D ratio significantly deteriorates the flight performance. Because further increases in charging power are difficult to achieve, the charging power decreases by 55.1% (54 kW), while the eUAV fleet size increases by 50.0% (1 fleet), the number of VCSs increases by 100.0% (1 VCS), and the number of chargers decreases by 33.3% (1 charger). The total system cost is increased by 43.4% ($393,015), verifying that designing eUAVs with low drag and high lift can significantly reduce the total system cost.
3. *Changes in specific energy density of battery*. With the development of battery technology, specific energy is gradually increasing. To account for advancements in future battery technology, optimization is conducted considering a 30% increase in specific energy density. The optimization results indicate that the eUAV fleet size and VCS design remain unchanged. Although the battery capacity decreases substantially by 38.1% (16 kWh), the flying range decreases by only 15.7% (4.2 km), and this reduction is offset by a modest 2.0% (2 kW) increase in charging power. Consequently, the total system cost decreases by 4.1% ($37,167), confirming that increasing the specific energy density effectively reduces aircraft weight and contributes to lowering the total system cost.

## Conclusion

In the near future, eRTs and eUAVs are expected to play a key role in addressing urban mobility challenges. While extensive research exists on eRT system optimization, studies on eUAV system design for human transportation remain limited, and comparative analyses of both systems are scarce. Therefore, this study presents a comparative optimization framework for eRT and eUAV systems, optimizing fleet operation, charging infrastructure design, and battery capacity design to minimize total system cost while satisfying passenger travel time constraints. The optimization results show that the eRT system maintains a lower total system cost than the eUAV system at higher target travel times due to its lower infrastructure requirements. However, as the target travel time decreases, the total cost of the eRT system increases sharply, and no feasible solution is found when the target travel time falls below 21 min. In contrast, the eUAV system remains viable even under shorter travel time conditions, demonstrating greater flexibility and scalability for fast urban transport. Although the eUAV system provides shorter average travel times, it exhibits higher variability and a greater likelihood of service delays compared to the eRT system. The design analysis reveals that VCS installation and maintenance costs are significantly higher than those of CSs, leading to an increase in charging power to minimize the number of VCSs. The number of eUAV fleets is much smaller than that of eRT fleets, indicating higher transport capability of eUAVs, while the larger battery capacity of eUAVs compared to eRTs can be attributed to their shorter flying range per battery capacity. As the target travel time decreases, the service distance and electricity cost of the eRT system increase sharply due to the larger fleet size and reduced energy efficiency. Parametric studies on eUAV weight, L/D ratio, and specific energy density of battery highlight their impact on system design and cost. An increase in eUAV weight or a reduction in the L/D ratio raises overall energy demand,^42^ requiring greater battery capacity to maintain operational performance. In contrast, improvements in battery specific energy density increase the flying range achievable for a given battery capacity and reduce the total system cost.

This study is novel in that it proposes a design framework for eUAV systems and successfully implements eUAV fleet operations within transportation systems. To the best of the authors’ knowledge, this study is one of the first to comprehensively compare eRT and eUAV systems in terms of cost and performance, addressing a critical gap in transportation research. There are several limitations in this study. The fidelity of the eUAV model should be improved by considering more specific engineering factors related to the flight of eUAVs. In addition, alternative conditions—such as different demand distributions and the inclusion or exclusion of passenger pooling—may yield different outcomes. Since the findings are based on Sejong City, a smart city with specific infrastructure characteristics, the results may not be directly generalizable to cities with different urban layouts or socioeconomic conditions. Finally, a detailed analysis of regulatory, safety, and public acceptance challenges should be conducted in the context of real-world adoption.

## Appendix

The equations and details for the eUAV model.

In the eUAV model, the flying range is obtained from a simplified energy-per-meter (EPM) formulation. The battery must supply both propulsion power to overcome weight and aerodynamic drag in level flight and avionics power for sensors, onboard processors, and communication. Given the battery capacity and the EPM value, the maximum flying range is calculated and then used in the main optimization model as a constraint on feasible missions. Equations for the eUAV model are given in Table A.1, and the parameters and symbols used in the eUAV model are listed in Table A.2.

**Table 9:** A.1 Equations for the eUAV model.

Equation	Expression	Description
(A.1)	${E_{{\mathrm{Propulsion}}}}=\int_{0}^{{{t_f}}} {\frac{{{m_a}(t)g{v_c}(t)}}{{r\eta }}dt}$	Propulsion energy required to overcome weight and drag and maintain level flight.
(A.2)	${E_{{\mathrm{Avionics}}}}=\int_{0}^{{{t_f}}} {{P_A}(t)dt}$	Electrical energy consumed by avionics and onboard electronics.
(A.3)	${E_{{\mathrm{Battery}}}}=\int_{0}^{{{t_f}}} {\left( {\frac{{{m_a}(t)g{v_c}(t)}}{{r\eta }}+{P_A}(t)} \right)dt}$	Total energy drawn from the battery.
(A.4)	$EPM=\frac{{{E_{{\mathrm{Battery}}}}}}{D}=\frac{{\int_{0}^{{{t_f}}} {\left( {\frac{{{m_a}(t)g{v_c}(t)}}{{r\eta }}+{P_A}(t)} \right)dt} }}{{\int_{0}^{{{t_f}}} {{v_c}(t)dt} }}$	Energy per meter of flight.

**Table 10:** A.2 Parameters and symbols used in the eUAV model.

Symbol	Meaning	Baseline value	unit
*t*_*f*_	Total flight time	−	s
*m*_*p*_	Mass of payload	75	kg
*m*_*s*_	Mass of eUAV body	85	kg
*m*_*b*_	mass of battery	−	kg
*m*_*a*_	Total mass of aircraft (*m*_*p*_*+m*_*s*_*+m*_*b*_)	−	kg
*v*_*c*_	Cruising speed	28.1	m/s
*g*	Gravitational acceleration	9.81	m/s^2^
*r*	Lift-to-drag (L/D) ratio	3	−
$\eta$	Battery-to-thrust power transfer efficiency	0.7	−
*P*_*A*_	Power demand of avionics and onboard electronics	500	W
*D*	Total flight distance	−	m

## Data Availability

The datasets used and/or analyzed during the current study available from the corresponding author on reasonable request.

## Code availability

The source code used in this work can be retrieved from the following link: 10.6084/m9.figshare.31403376, only for further analysis by the readers.

## References

[1] Lee, U., Jeon, S. & Lee, I. Design for shared autonomous vehicle (SAV) system employing electrified vehicles: Comparison of battery electric vehicles (BEVs) and fuel cell electric vehicles (FCEVs). *Clean. Eng. Technol.* **8**, 100505 (2022).

[2] Wang, N. et al. Cost-oriented optimization of the location and capacity of charging stations for the electric robotaxi fleet. *Energy* **263**, 125895 (2023).

[3] Farhan, J. & Chen, T. D. Impact of ridesharing on operational efficiency of shared autonomous electric vehicle fleet. *Transp. Res. Part. C Emerg. Technol.* **93**, 310–321 (2018).

[4] Riccardo, I., Benjamin, L. & Tetsuo, T. Modeling shared autonomous electric vehicles: Potential for transport and power grid integration. *Energy* **158**, 148–163 (2018).

[5] Vosooghi, R., Puchinger, J., Bischoff, J., Jankovic, M. & Vouillon, A. Shared autonomous electric vehicle service performance: Assessing the impact of charging infrastructure. *Transp. Res. Part. D Transp. Environ.* **81**, 102283 (2020).

[6] Chen, T. M., Kockelman, K. M. & Hanna, J. P. Operations of a shared, autonomous, electric vehicle fleet: Implications of vehicle and charging infrastructure decisions. *Transp. Res. Part. Policy Pract.* **94**, 243–254 (2016).

[7] Kim, S., Lee, U., Lee, I. & Kang, N. Idle vehicle relocation strategy through deep learning for shared autonomous electric vehicle system optimization. *J. Clean. Prod.* **333**, 130055 (2022).

[8] Lee, U., Kang, N. & Lee, Y. K. Shared autonomous electric vehicle system design and optimization under dynamic battery degradation considering varying load conditions. *J. Clean. Prod.* **423**, 138795 (2023).

[9] Al-Kaff, A., Martin, D., Garcia, F., de la Escalera, A. & Armingol, J. M. Survey of computer vision algorithms and applications for unmanned aerial vehicles. *Expert Syst. Appl.* **92**, 447–463 (2018).

[10] Saeed, A. S., Younes, A. B., Cai, C. & Cai, G. A survey of hybrid unmanned aerial vehicles. *Prog Aerosp. Sci.* **98**, 91–105 (2018).

[11] Xiao, C., Wang, B., Zhao, D. & Wang, C. Comprehensive investigation on lithium batteries for electric and hybrid-electric unmanned aerial vehicle applications. *Therm. Sci. Eng. Prog*. **38**, 101677 (2023).

[12] Pan, Z., An, L. & Wen, C. Recent advances in fuel cells-based propulsion systems for unmanned aerial vehicles. *Appl. Energy*. **240**, 473–485 (2019).

[13] Zhang, K., Lu, L., Lei, C., Zhu, H. & Ouyang, Y. Dynamic operations and pricing of electric unmanned aerial vehicle systems and power networks. *Transp. Res. Part. C Emerg. Technol.* **92**, 472–485 (2018).

[14] Luo, Y. W., Qian, Y. P., Zeng, Z. Z. & Zhang, Y. J. Simulation and analysis of operating characteristics of power battery for flying car utilization. *eTransportation* **8**, 100111 (2021).

[15] Michel, N., Wei, P., Kong, Z., Sinha, A. K. & Lin, X. Modeling and validation of electric multirotor unmanned aerial vehicle system energy dynamics. *eTransportation* **12**, 100173 (2022).

[16] Choi, J. & Kim, H. M. State-of-the-art of Korean smart cities: A critical review of the Sejong smart city plan. In: Smart Cities Technology and Social Innovation, Elsevier, pp 51–72 (2021).

[17] NAVER Map. Real-time traffic. (2024). https://map.naver.com/ (Accessed June 18, 2024).

[18] Urban Traffic Information Center. Open data. (2024). https://www.utic.go.kr/guide/newUtisData.do (Accessed July 11, 2024).

[19] Dijkstra, E. W. A note on two problems in connexion with graphs. *Numer. Math.* **1**, 269–271 (1959).

[20] Haris, F. et al. A review of the plantar pressure distribution effects from insole materials and at different walking speeds. *Appl. Sci.* **11**, 11851 (2021).

[21] Kang, N., Feinberg, F. M. & Papalambros, P. Y. Autonomous electric vehicle sharing system design. *J. Mech. Des.* **139** (1), 011402 (2017).

[22] Kchaou-Boujelben, M. Charging station location problem: A comprehensive review on models and solution approaches. *Transp. Res. Part. C Emerg. Technol.* **132**, 103376 (2021).

[23] Lee, U. A new adaptive Kriging-based optimization (AKBO) framework for constrained optimization problems: A case study on shared autonomous electric vehicle system design. *Expert Syst. Appl.* **252**, 124147 (2024).

[24] Borlaug, B., Salisbury, S., Gerdes, M. & Muratori, M. Levelized cost of charging electric vehicles in the United States. *Joule* **4** (7), 1470–1485 (2020).

[25] Chang, S. E. & Woo, J. Are electric vehicle users willing to pay tax for charging electric vehicles? A case study of South Korea. *Energy Econ.* **129**, 107243 (2024).

[26] Lee, U., Kang, N. & Lee, I. Selection of optimal target reliability in RBDO through reliability-based design for market systems (RBDMS) and application to electric vehicle design. *Struct. Multidisc Optim.* **60** (3), 949–963 (2019).

[27] Valdes, R. How much are electric cars? (2024). https://www.kbb.com/car-advice/how-much-electric-car-cost/ (Accessed August 8, 2024).

[28] The Mack Institute. Analysis shows continued industry-wide decline in electric vehicle battery costs. (2017). https://mackinstitute.wharton.upenn.edu/2018/electric-vehicle-battery-costs-decline/ (Accessed July 15, 2024).

[29] Lee, U., Kang, N. & Lee, I. Shared autonomous electric vehicle design and operations under uncertainties: A reliability-based design optimization approach. *Struct. Multidisc Optim.* **61**, 1529–1545 (2020).

[30] Yoon, M., Park, J., Park, B., Jin, T. & Choo, H. Study of optimal base station deployment for UAM operations in an urban environment based on a genetic algorithm. *IEEE Access.* **13**, 127570–127579 (2025).

[31] Hyundai Elevator. Passenger elevators https:// (2024). www.hyundaielevator.co.kr/upload/product-catalog/2402_Passenger%20Elevator_LUXEN%20n%20NEW%20YZER(1).pdf (Accessed October 30, 2025).

[32] Fukumine, Y. & Lei, Z. Estimation of eVTOL flight performance using rotorcraft theory. In: The 33rd Congress of the International Council of the Aeronautical Science, 1–9 (2022).

[33] Aviation Week Network. Skyportz CEO describes modular ‘Vertiport-In-A-Box’ concept. (2023). https://aviationweek.com/aerospace/advanced-air-mobility/skyportz-ceo-describes-modular-vertiport-box-concept (Accessed July 1, 2024).

[34] D’Andrea, R. Guest editorial: Can drones deliver? *IEEE Trans. Autom. Sci. Eng.* **11** (3), 647–648 (2014).

[35] Memon, O. Tiny personal eVTOL: A look at Jetson One. (2024). https://simpleflying.com/jetson-one-personal-evtol-guide/ (Accessed August 10, 2024).

[36] Menzi, D. et al. Ultra-lightweight high-efficiency buck-boost DC–DC converters for future eVTOL aircraft with hybrid power supply. *IEEE Trans. Transp. Electrific*. **10** (4), 10297–10313 (2024).

[37] Ramirez, V. B. This tiny personal aircraft costs under $100K and can take off from your driveway. (2021). https://singularityhub.com/2021/10/27/this-tiny-personal-aircraft-costs-under-100k-and-can-take-off-from-your-driveway/ (Accessed June 29, 2024).

[38] Ministry of Data and Statistics. Consumer price survey (2025). https://kostat.go.kr/board.es?mid=a20109020000&bid=11751 (Accessed October 29, 2025).

[39] Mohammed, A., Saif, O., Abo-Adma, M., Fahmy, A. & Elazab, R. Strategies and sustainability in fast charging station deployment for electric vehicles. *Sci. Rep.* **14** (1), 283 (2024). 38168937 10.1038/s41598-023-50825-7 PMC10762045

[40] Ramachandaramurthy, V. K. et al. Social acceptance and preference of EV users—a review. *IEEE Access.* **11**, 11956–11972 (2023).

[41] Starkey, R. P. & Lewis, M. J. Analytical off-design lift-to-drag ratio analysis for hypersonic waveriders. *J. Spacecr. Rockets*. **37** (5), 684–691 (2000).

[42] Lee, Y. K., Lee, U. & Kang, N. Multi-scale design optimization of electric vehicles by analytical target cascading: From battery cell level to marketing level. *J. Clean. Prod.* **368**, 133235 (2022).

